# 2-(Pyridin-4-yl)-2,3-di­hydro-1*H*-naphtho­[1,8-*de*][1,3,2]di­aza­borinine

**DOI:** 10.1107/S2414314624006151

**Published:** 2024-06-28

**Authors:** Ryo Yamamoto, Shu Hashimoto, Tsunehisa Okuno

**Affiliations:** a Department of Systems Engineering Wakayama University, Sakaedani Wakayama 640-8510 Japan; University of Antofagasta, Chile

**Keywords:** crystal structure, pyridine derivative, tetra­meric structure, dan

## Abstract

The title compound, C_15_H_12_BN_3_, is a type of di­aza­borinane featuring substitution at 1, 2, and 3 positions in the nitro­gen–boron six-membered heterocycle. It is comprised of two almost planar units, the pyridyl ring and the Bdan (dan = 1,8-di­aminona­phtho) group, which subtend a dihedral angle of 24.57 (5)°. In the crystal, the mol­ecules are linked into *R*_4_^4^(28) hydrogen-bonding networks around the fourfold inversion axis, giving cyclic tetra­mers. The mol­ecules form columnar stacks along the *c* axis.

## Abstract 2

In the title compound, the pyridyl ring and the Bdan (dan = 1,8-di­aminona­phto) group subtend a dihedral angle of 24.57 (5)°. In the crystal, the mol­ecules make 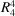
(28) hydrogen-bonding networks around the fourfold inversion axis, giving a cyclic tetra­mer. The mol­ecules form columnar stacks along the *c* axis.

## Graphical abstract

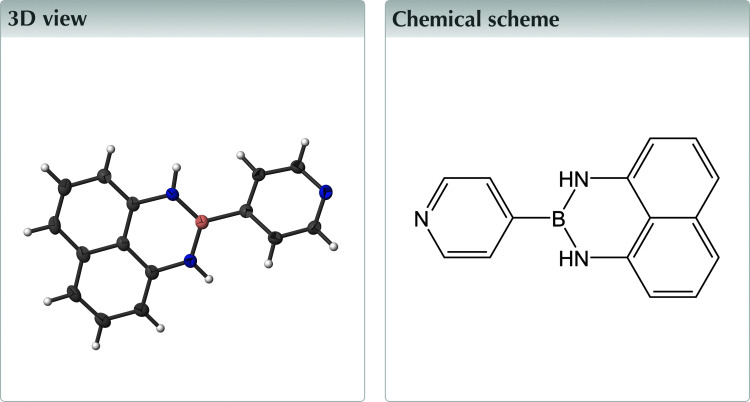

## Structure description

The title compound, C_15_H_12_BN_3_, is a type of di­aza­borinane that is substituted at the 1, 2, and 3 positions in the nitro­gen–boron six-membered heterocycle. Recently, di­aza­borinanes have been found to stabilize organic radicals (LaPorte *et al.*, 2023 ▸).

**Figure 1:**
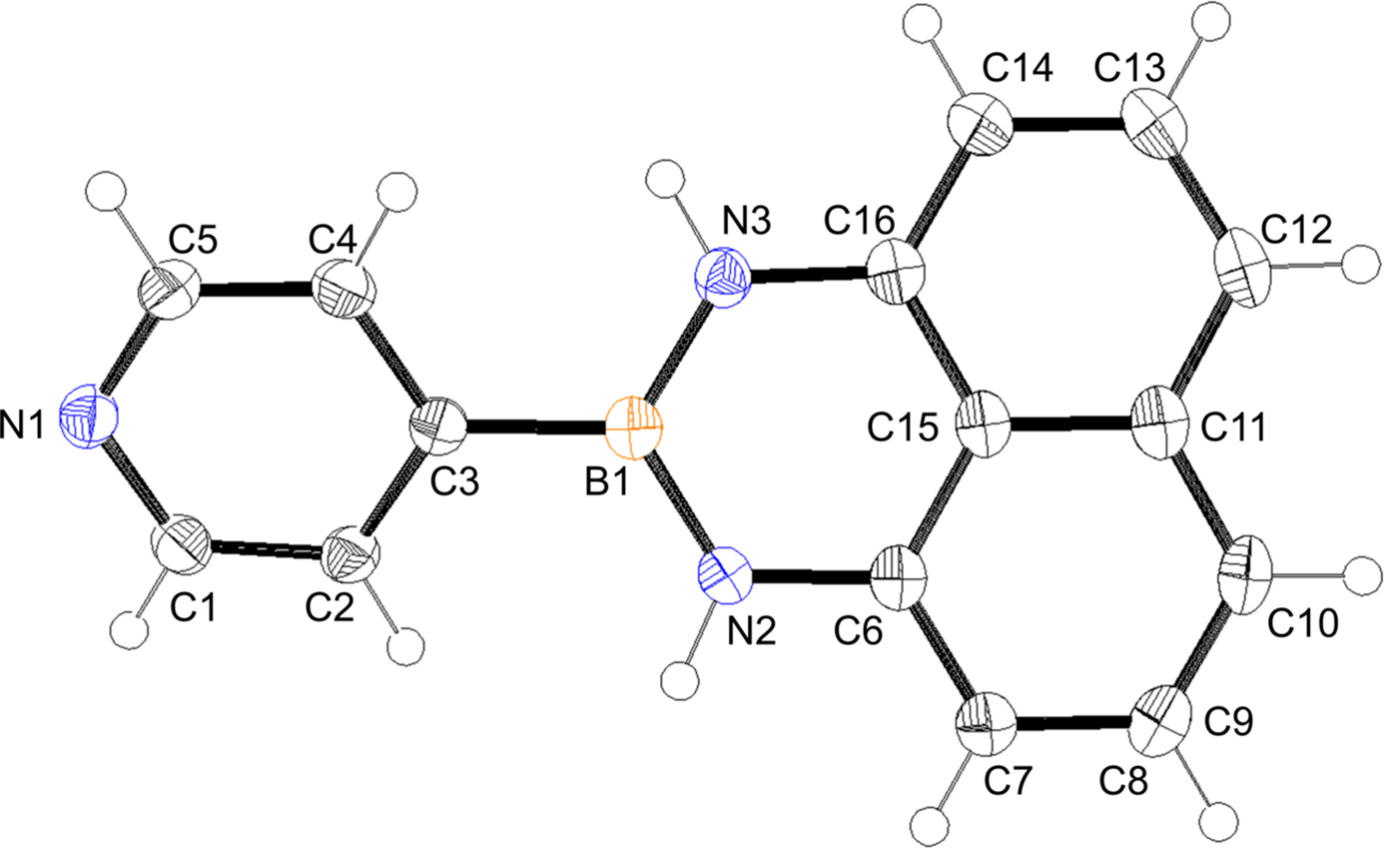
The title compound with the atom-numbering scheme. Displacement ellipsoids are drawn at the 50% probability level and H atoms are shown as small spheres of arbitrary radii.

The title mol­ecule (Fig. 1 ▸) is comprised of two almost planar units, the N1/C1–C5 pyridyl ring and the N2/N3/C6–C15/B1 group, which subtend a dihedral angle of 24.57 (5)°. This is slightly larger than those in related compounds that have almost planar structures (Akerman *et al.*, 2011 ▸; Slabber *et al.*, 2011 ▸).

**Figure 2:**
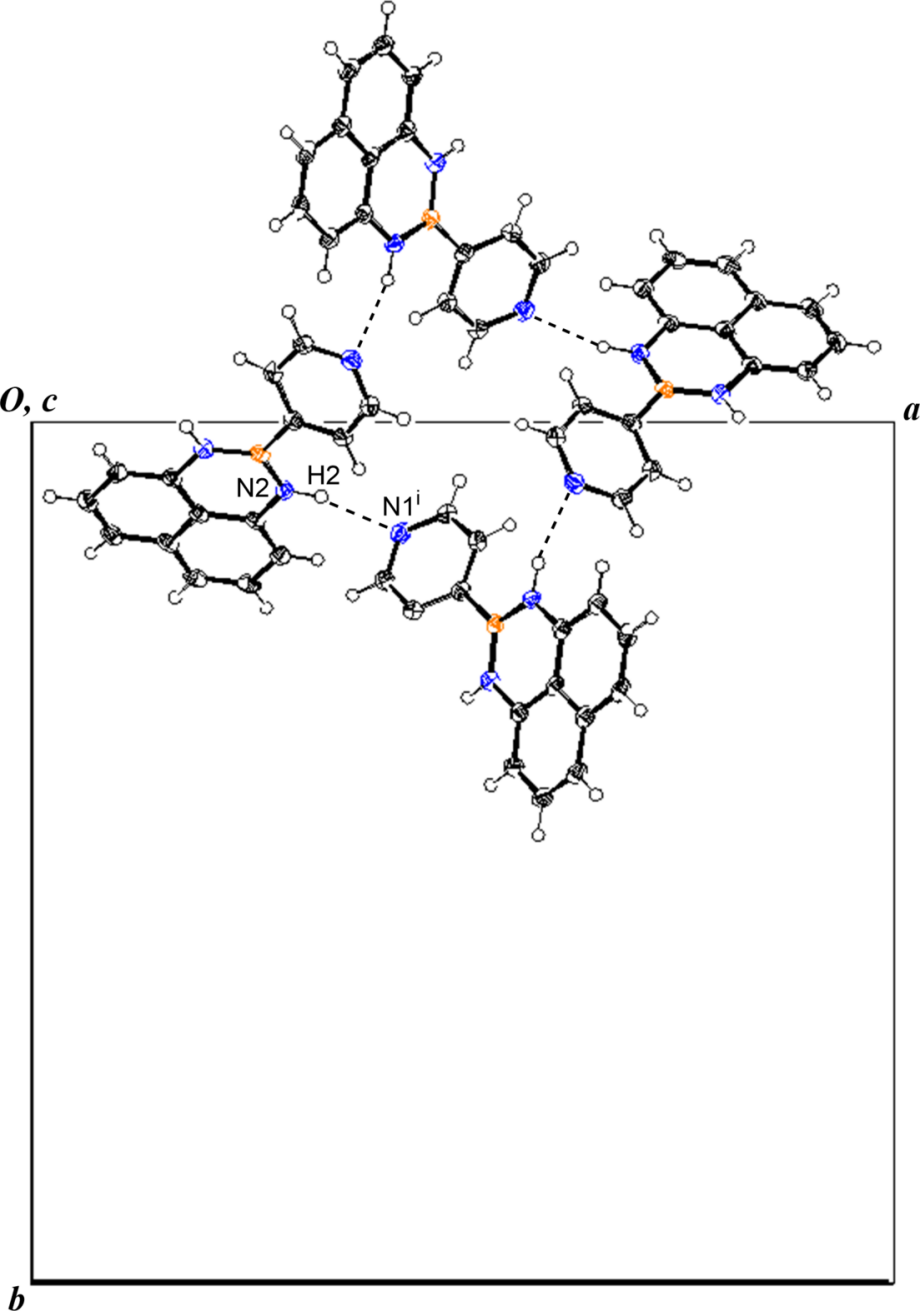
The hydrogen-bonding network of the title compound. [Symmetry code: (i) *y* + 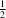, −*x* + 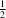, −*z* − 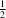.]

**Figure 3:**
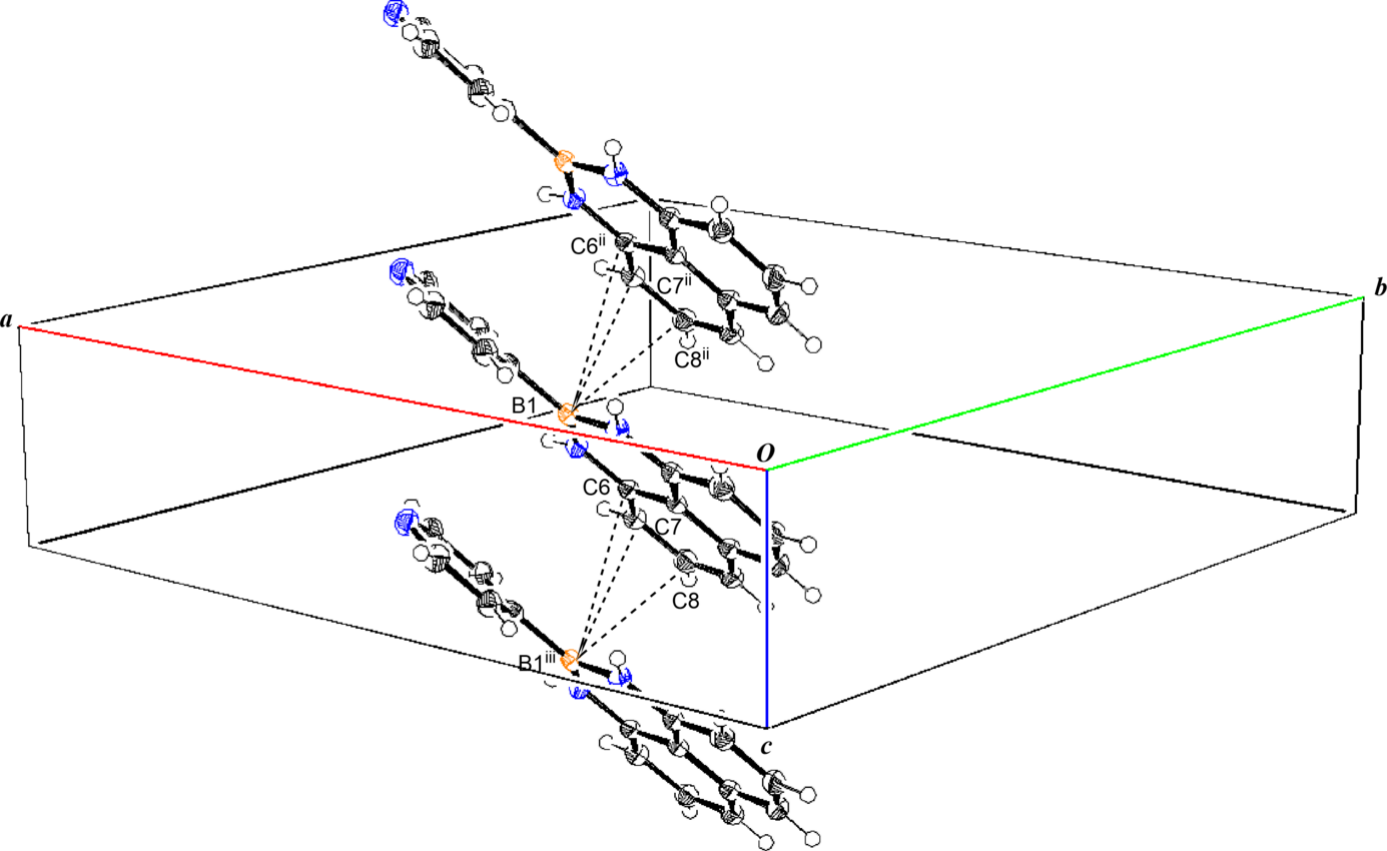
The stacking structure along the *c* axis. [Symmetry codes:(ii) *x*, *y*, *z* − 1; (iii) *x*, *y*, *z* + 1.]

In the crystal, the mol­ecules make 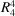
(28) hydrogen-bonding (Table 1 ▸) networks around the fourfold inversion axis, giving a cyclic tetra­mer as shown in Fig. 2 ▸. The formation of this tetra­meric structure is thought to increase the dihedral angle. The mol­ecules also stack along the *c* axis, as shown in Fig. 3 ▸, forming columnar stacks in which the B1⋯C6^ii^, B1⋯C7^ii^, and B1⋯C8^ii^ distances are 3.656 (3), 3.513 (3) and 3.573 (3) Å, respectively [symmetry code:(ii) *x*, *y*, *z* − 1].

## Synthesis and crystallization

The title compound was prepared according to the literature method (Hashimoto & Okuno, 2024 ▸). Single crystals of sufficient quality were obtained by recrystallization from chloro­form solution.

## Refinement

Crystal data, data collection and structure refinement details are summarized in Table 2 ▸.

## Supplementary Material

Crystal structure: contains datablock(s) I. DOI: 10.1107/S2414314624006151/bx4031sup1.cif

Structure factors: contains datablock(s) I. DOI: 10.1107/S2414314624006151/bx4031Isup2.hkl

Supporting information file. DOI: 10.1107/S2414314624006151/bx4031Isup3.cml

CCDC reference: 2364937

Additional supporting information:  crystallographic information; 3D view; checkCIF report

## Figures and Tables

**Table 1:** Hydrogen-bond geometry (Å, °)

*D*—H⋯*A*	*D*—H	H⋯*A*	*D*⋯*A*	*D*—H⋯*A*
N2—H2⋯N1^i^	0.93 (2)	2.21 (2)	3.113 (2)	162 (2)

Symmetry code: (i) 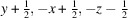.

**Table 2:** Experimental details

Crystal data	
Chemical formula	C_15_H_12_BN_3_
*M*_r_	245.09
Crystal system, space group	Tetragonal, *I* 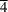
Temperature (K)	100
*a*, *c* (Å)	21.5659 (3), 5.0863 (1)
*V* (Å^3^)	2365.58 (8)
*Z*	8
Radiation type	Cu *K*α
μ (mm^−1^)	0.65
Crystal size (mm)	0.20 × 0.05 × 0.05

Data collection	
Diffractometer	XtaLAB Synergy R, DW system, HyPix
Absorption correction	Multi-scan (*CrysAlis PRO*; Rigaku OD, 2024 ▸)
*T*_min_, *T*_max_	0.807, 1.000
No. of measured, independent and observed [*I* > 2σ(*I*)] reflections	7772, 2268, 2125
*R*_int_	0.036
(sin θ/λ)_max_ (Å^−1^)	0.626

Refinement	
*R*[*F*^2^ > 2σ(*F*^2^)], *wR*(*F*^2^), *S*	0.031, 0.083, 1.06
No. of reflections	2268
No. of parameters	180
H-atom treatment	H atoms treated by a mixture of independent and constrained refinement
Δρ_max_, Δρ_min_ (e Å^−3^)	0.15, −0.16
Absolute structure	Flack *x* determined using 840 quotients [(*I*^+^)−(*I*^−^)]/[(*I*^+^)+(*I*^−^)] (Parsons *et al.*, 2013 ▸)
Absolute structure parameter	−0.2 (3)

Computer programs: *CrysAlis PRO* (Rigaku OD, 2024 ▸), *SHELXS* (Sheldrick, 2008 ▸), *SHELXL2013/2* (Sheldrick, 2015 ▸) and *OLEX2* (Dolomanov *et al.*, 2009 ▸).

## full crystallographic data

### \ 2-(Pyridin-4-yl)-2,3-dihydro-1H-naphtho[1,8-de][1,3,2]\ diazaborinine Crystal data

**Table:**

C_15_H_12_BN_3_	*D*_x_ = 1.376 Mg m^−^^3^
*M**_r_* = 245.09	Cu *K*α radiation, λ = 1.54184 Å
Tetragonal, *I*4	Cell parameters from 5132 reflections
*a* = 21.5659 (3) Å	θ = 2.9–75.0°
*c* = 5.0863 (1) Å	µ = 0.65 mm^−^^1^
*V* = 2365.58 (8) Å^3^	*T* = 100 K
*Z* = 8	Block, clear colourless
*F*(000) = 1024	0.2 × 0.05 × 0.05 mm

### \ 2-(Pyridin-4-yl)-2,3-dihydro-1H-naphtho[1,8-de][1,3,2]\ diazaborinine Data collection

**Table:**

XtaLAB Synergy R, DW system, HyPix diffractometer	2268 independent reflections
Radiation source: Rotating-anode X-ray tube, Rigaku (Cu) X-ray Source	2125 reflections with *I* > 2σ(*I*)
Mirror monochromator	*R*_int_ = 0.036
Detector resolution: 10.0000 pixels mm^-1^	θ_max_ = 75.0°, θ_min_ = 2.9°
ω scans	*h* = −25→26
Absorption correction: multi-scan (CrysAlisPro; Rigaku OD, 2024)	*k* = −26→27
*T*_min_ = 0.807, *T*_max_ = 1.000	*l* = −5→6
7772 measured reflections	

### \ 2-(Pyridin-4-yl)-2,3-dihydro-1H-naphtho[1,8-de][1,3,2]\ diazaborinine Refinement

**Table:**

Refinement on *F*^2^	Hydrogen site location: mixed
Least-squares matrix: full	H atoms treated by a mixture of independent and constrained refinement
*R*[*F*^2^ > 2σ(*F*^2^)] = 0.031	*w* = 1/[σ^2^(*F*_o_^2^) + (0.0502*P*)^2^ + 0.1789*P*] where *P* = (*F*_o_^2^ + 2*F*_c_^2^)/3
*wR*(*F*^2^) = 0.083	(Δ/σ)_max_ < 0.001
*S* = 1.06	Δρ_max_ = 0.15 e Å^−^^3^
2268 reflections	Δρ_min_ = −0.15 e Å^−^^3^
180 parameters	Absolute structure: Flack *x* determined using 840 quotients [(*I*^+^)-(*I*^-^)]/[(*I*^+^)+(*I*^-^)] (Parsons *et al.*, 2013)
0 restraints	Absolute structure parameter: −0.2 (3)
Primary atom site location: structure-invariant direct methods	

### \ 2-(Pyridin-4-yl)-2,3-dihydro-1H-naphtho[1,8-de][1,3,2]\ diazaborinine Special details

**Table:**

Geometry. All esds (except the esd in the dihedral angle between two l.s. planes) are estimated using the full covariance matrix. The cell esds are taken into account individually in the estimation of esds in distances, angles and torsion angles; correlations between esds in cell parameters are only used when they are defined by crystal symmetry. An approximate (isotropic) treatment of cell esds is used for estimating esds involving l.s. planes.
Refinement. The positions of the N-bound H atoms were obtained from difference Fourier maps and were refined isotropically. The C-bound H atoms were placed at ideal positions and were refined as riding on their parent C atoms. *U*_iso_(H) values of the H atoms were set at 1.2*U*_eq_(parent atom for C*sp*^2^).

### \ 2-(Pyridin-4-yl)-2,3-dihydro-1H-naphtho[1,8-de][1,3,2]\ diazaborinine Fractional atomic coordinates and isotropic or equivalent isotropic displacement parameters (Å^2^)

**Table:**

	*x*	*y*	*z*	*U*_iso_*/*U*_eq_	
N3	0.20041 (7)	0.03150 (8)	−0.0041 (4)	0.0233 (4)	
C7	0.28868 (9)	0.15497 (9)	0.5132 (4)	0.0252 (4)	
H7	0.3324	0.1604	0.5071	0.030*	
C1	0.39265 (9)	−0.01777 (9)	−0.5063 (4)	0.0270 (4)	
H1	0.4315	−0.0032	−0.5689	0.032*	
N2	0.29394 (8)	0.07934 (7)	0.1587 (3)	0.0217 (3)	
C13	0.10022 (9)	0.05895 (9)	0.1820 (4)	0.0267 (4)	
H13	0.0797	0.0324	0.0611	0.032*	
C10	0.15895 (9)	0.14042 (9)	0.5376 (4)	0.0255 (4)	
C15	0.19455 (9)	0.10642 (8)	0.3491 (4)	0.0219 (4)	
C5	0.31573 (9)	−0.08947 (9)	−0.5129 (4)	0.0273 (4)	
H5	0.2989	−0.1270	−0.5796	0.033*	
C6	0.26012 (9)	0.11421 (8)	0.3414 (4)	0.0218 (4)	
C14	0.16387 (9)	0.06513 (9)	0.1728 (4)	0.0231 (4)	
C11	0.09363 (10)	0.13134 (9)	0.5452 (4)	0.0288 (5)	
H11	0.0693	0.1527	0.6721	0.035*	
B1	0.26579 (10)	0.03753 (10)	−0.0181 (4)	0.0220 (4)	
C3	0.30411 (9)	−0.00146 (9)	−0.2242 (4)	0.0220 (4)	
C4	0.28147 (9)	−0.05701 (9)	−0.3271 (4)	0.0262 (4)	
H4	0.2426	−0.0727	−0.2700	0.031*	
C12	0.06564 (9)	0.09191 (9)	0.3700 (4)	0.0286 (5)	
H12	0.0219	0.0868	0.3758	0.034*	
N1	0.37087 (8)	−0.07118 (8)	−0.6036 (3)	0.0268 (4)	
C2	0.36184 (9)	0.01756 (9)	−0.3191 (4)	0.0246 (4)	
H2A	0.3801	0.0547	−0.2555	0.030*	
C9	0.19006 (10)	0.18127 (9)	0.7109 (4)	0.0277 (5)	
H9	0.1670	0.2039	0.8380	0.033*	
C8	0.25310 (10)	0.18841 (9)	0.6969 (4)	0.0279 (4)	
H8	0.2732	0.2164	0.8132	0.033*	
H3	0.1804 (12)	0.0058 (12)	−0.125 (6)	0.042 (7)*	
H2	0.3364 (11)	0.0870 (10)	0.167 (5)	0.028 (6)*	

### \ 2-(Pyridin-4-yl)-2,3-dihydro-1H-naphtho[1,8-de][1,3,2]\ diazaborinine Atomic displacement parameters (Å^2^)

**Table:**

	*U*^11^	*U*^22^	*U*^33^	*U*^12^	*U*^13^	*U*^23^
N3	0.0236 (8)	0.0238 (8)	0.0225 (8)	0.0017 (6)	−0.0004 (7)	−0.0003 (7)
C7	0.0272 (9)	0.0252 (9)	0.0233 (10)	0.0016 (8)	−0.0001 (8)	0.0025 (8)
C1	0.0229 (9)	0.0263 (9)	0.0317 (10)	0.0004 (8)	0.0016 (9)	0.0004 (9)
N2	0.0203 (8)	0.0225 (7)	0.0223 (8)	0.0012 (6)	0.0012 (7)	0.0008 (7)
C13	0.0254 (10)	0.0260 (9)	0.0287 (10)	−0.0001 (8)	0.0002 (9)	0.0042 (9)
C10	0.0313 (10)	0.0235 (9)	0.0216 (10)	0.0058 (8)	0.0042 (8)	0.0068 (8)
C15	0.0262 (9)	0.0202 (8)	0.0193 (9)	0.0045 (7)	0.0022 (8)	0.0053 (7)
C5	0.0272 (10)	0.0245 (9)	0.0301 (10)	−0.0008 (8)	−0.0020 (9)	−0.0043 (8)
C6	0.0270 (9)	0.0200 (8)	0.0183 (9)	0.0027 (7)	0.0016 (8)	0.0050 (8)
C14	0.0271 (9)	0.0209 (9)	0.0214 (9)	0.0025 (7)	0.0018 (8)	0.0057 (8)
C11	0.0301 (10)	0.0283 (10)	0.0278 (11)	0.0088 (8)	0.0094 (9)	0.0068 (8)
B1	0.0254 (10)	0.0194 (9)	0.0212 (10)	0.0027 (8)	0.0001 (9)	0.0041 (9)
C3	0.0227 (9)	0.0228 (9)	0.0204 (9)	0.0030 (7)	−0.0033 (7)	0.0017 (7)
C4	0.0223 (9)	0.0274 (9)	0.0290 (10)	−0.0008 (8)	−0.0003 (8)	0.0000 (9)
C12	0.0248 (9)	0.0290 (10)	0.0319 (11)	0.0044 (8)	0.0051 (9)	0.0092 (9)
N1	0.0272 (9)	0.0263 (8)	0.0270 (9)	0.0038 (7)	−0.0015 (7)	−0.0034 (7)
C2	0.0253 (9)	0.0210 (9)	0.0276 (10)	−0.0009 (8)	−0.0009 (8)	−0.0012 (8)
C9	0.0383 (11)	0.0243 (10)	0.0205 (10)	0.0092 (8)	0.0042 (9)	0.0014 (8)
C8	0.0387 (11)	0.0223 (9)	0.0226 (10)	0.0035 (8)	−0.0019 (9)	0.0010 (8)

### \ 2-(Pyridin-4-yl)-2,3-dihydro-1H-naphtho[1,8-de][1,3,2]\ diazaborinine Geometric parameters (Å, º)

**Table:**

N3—C14	1.399 (3)	C10—C9	1.416 (3)
N3—B1	1.418 (3)	C15—C6	1.425 (3)
N3—H3	0.93 (3)	C15—C14	1.426 (3)
C7—H7	0.9500	C5—H5	0.9500
C7—C6	1.384 (3)	C5—C4	1.389 (3)
C7—C8	1.408 (3)	C5—N1	1.335 (3)
C1—H1	0.9500	C11—H11	0.9500
C1—N1	1.339 (3)	C11—C12	1.372 (3)
C1—C2	1.389 (3)	B1—C3	1.578 (3)
N2—C6	1.401 (2)	C3—C4	1.396 (3)
N2—B1	1.411 (3)	C3—C2	1.397 (3)
N2—H2	0.93 (2)	C4—H4	0.9500
C13—H13	0.9500	C12—H12	0.9500
C13—C14	1.380 (3)	C2—H2A	0.9500
C13—C12	1.405 (3)	C9—H9	0.9500
C10—C15	1.431 (3)	C9—C8	1.370 (3)
C10—C11	1.423 (3)	C8—H8	0.9500
			
C14—N3—B1	123.02 (17)	C13—C14—N3	122.15 (18)
C14—N3—H3	118.1 (17)	C13—C14—C15	120.03 (18)
B1—N3—H3	118.8 (17)	C10—C11—H11	119.9
C6—C7—H7	119.9	C12—C11—C10	120.23 (18)
C6—C7—C8	120.13 (18)	C12—C11—H11	119.9
C8—C7—H7	119.9	N3—B1—C3	120.36 (18)
N1—C1—H1	118.0	N2—B1—N3	117.04 (18)
N1—C1—C2	123.90 (18)	N2—B1—C3	122.60 (17)
C2—C1—H1	118.0	C4—C3—B1	121.57 (17)
C6—N2—B1	122.82 (16)	C4—C3—C2	115.74 (18)
C6—N2—H2	112.8 (15)	C2—C3—B1	122.68 (17)
B1—N2—H2	124.4 (15)	C5—C4—C3	120.12 (18)
C14—C13—H13	119.9	C5—C4—H4	119.9
C14—C13—C12	120.1 (2)	C3—C4—H4	119.9
C12—C13—H13	119.9	C13—C12—H12	119.3
C11—C10—C15	118.62 (19)	C11—C12—C13	121.46 (18)
C9—C10—C15	118.83 (18)	C11—C12—H12	119.3
C9—C10—C11	122.54 (18)	C5—N1—C1	116.03 (17)
C6—C15—C10	119.37 (18)	C1—C2—C3	120.15 (17)
C6—C15—C14	121.14 (17)	C1—C2—H2A	119.9
C14—C15—C10	119.49 (17)	C3—C2—H2A	119.9
C4—C5—H5	118.0	C10—C9—H9	119.7
N1—C5—H5	118.0	C8—C9—C10	120.51 (19)
N1—C5—C4	124.06 (18)	C8—C9—H9	119.7
C7—C6—N2	121.89 (17)	C7—C8—H8	119.4
C7—C6—C15	119.97 (17)	C9—C8—C7	121.19 (19)
N2—C6—C15	118.14 (16)	C9—C8—H8	119.4
N3—C14—C15	117.81 (16)		
			
N3—B1—C3—C4	−23.9 (3)	C11—C10—C15—C6	−179.01 (17)
N3—B1—C3—C2	154.92 (19)	C11—C10—C15—C14	0.7 (3)
N2—B1—C3—C4	156.09 (19)	C11—C10—C9—C8	179.81 (19)
N2—B1—C3—C2	−25.1 (3)	B1—N3—C14—C13	179.50 (19)
C10—C15—C6—C7	−0.8 (3)	B1—N3—C14—C15	−1.1 (3)
C10—C15—C6—N2	178.65 (16)	B1—N2—C6—C7	179.99 (18)
C10—C15—C14—N3	−178.38 (17)	B1—N2—C6—C15	0.5 (3)
C10—C15—C14—C13	1.0 (3)	B1—C3—C4—C5	178.49 (18)
C10—C11—C12—C13	0.9 (3)	B1—C3—C2—C1	−178.13 (18)
C10—C9—C8—C7	−0.8 (3)	C4—C5—N1—C1	−0.6 (3)
C15—C10—C11—C12	−1.7 (3)	C4—C3—C2—C1	0.7 (3)
C15—C10—C9—C8	0.5 (3)	C12—C13—C14—N3	177.53 (17)
C6—C7—C8—C9	0.3 (3)	C12—C13—C14—C15	−1.8 (3)
C6—N2—B1—N3	−0.3 (3)	N1—C1—C2—C3	−1.1 (3)
C6—N2—B1—C3	179.77 (16)	N1—C5—C4—C3	0.3 (3)
C6—C15—C14—N3	1.4 (3)	C2—C1—N1—C5	0.9 (3)
C6—C15—C14—C13	−179.24 (18)	C2—C3—C4—C5	−0.4 (3)
C14—N3—B1—N2	0.6 (3)	C9—C10—C15—C6	0.3 (3)
C14—N3—B1—C3	−179.47 (17)	C9—C10—C15—C14	−179.93 (16)
C14—C13—C12—C11	0.9 (3)	C9—C10—C11—C12	179.00 (18)
C14—C15—C6—C7	179.45 (17)	C8—C7—C6—N2	−178.96 (17)
C14—C15—C6—N2	−1.1 (3)	C8—C7—C6—C15	0.5 (3)

### \ 2-(Pyridin-4-yl)-2,3-dihydro-1H-naphtho[1,8-de][1,3,2]\ diazaborinine Hydrogen-bond geometry (Å, º)

**Table:**

*D*—H···*A*	*D*—H	H···*A*	*D*···*A*	*D*—H···*A*
N2—H2···N1^i^	0.93 (2)	2.21 (2)	3.113 (2)	162 (2)

Symmetry code: (i) *y*+1/2, −*x*+1/2, −*z*−1/2.
